# Range reexpansion after long stasis: Italian otters (*Lutra lutra*) at their northern edge

**DOI:** 10.1002/ece3.9726

**Published:** 2023-01-04

**Authors:** Manlio Marcelli, Federico Striglioni, Romina Fusillo

**Affiliations:** ^1^ Lutria sas Wildlife Research and Consulting Rome Italy; ^2^ Gran Sasso and Monti della Laga National Park Assergi Italy

**Keywords:** Italy, *Lutra lutra*, range margin, range reexpansion, segmented regression, species range

## Abstract

Species range shifts and expansion are subjects of primary research interest in the context of climate warming and biological invasions. Few studies have focused on reexpansion of species that suffered severe declines. Here, we focused on population recovery of Eurasian otters (*Lutra lutra*) in Italy, first detected in 2003 after a southward range contraction. We modeled the rate of range expansion and occupancy at the northern expanding front (central Italy), to gain insights into the progress of recovery and mechanisms of reexpansion. We performed a field survey in 2021, which redefined the northern limit of distribution further north, in close proximity to the Gran Sasso National Park. Then we analyzed a time series (1985–2021) of distances of northernmost occurrences from the center of the 1985 range. Using segmented regression, we were able to identify a prolonged stasis of the northern range edge and a simultaneous increase in occupancy from 0.151 to 0.4. A breakpoint was estimated in 2006, after which the range expanded northwards at an average rate of 5.48 km/year. From 2006 to 2021, the overall northward shift was about 80 km. Occupancy continued to increase until 2019 and abruptly declined in 2021. These patterns suggest that the reexpansion of the range can be limited by low occupancy at the expanding front. As occupancy increases, long‐distance dispersal increases and then range expands. The low occupancy at the current distribution limit of otters may reflect a higher anthropogenic pressure on northern habitats, which could slow down the reexpansion process.

## Graphical abstract

The paper focuses on range reexpansion of the endangered Eurasian otter (*Lutra lutra*) in Italy. We modeled the rate of range expansion at the northern expanding front (central Italy) to gain insights into mechanisms of species reexpansion. We analyzed a time series of occupancy rates and distance measures on northernmost occurrences of the species (1985–2021). On the premise that the reexpansion of a species is a non‐linear process, we were able to identify the onset of reexpansion and test for different model forms underlying different hypotheses using segmented regression. The main results support the hypothesis that range reexpansion starts when the occupancy rate at the expanding front reaches a threshold value.

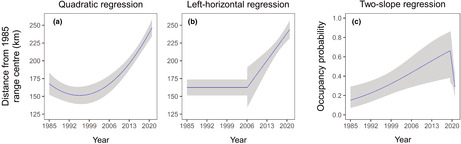

## 1 INTRODUCTION

Species range contraction and expansion driven by climate and land use changes, and biological invasions are increasingly important study areas (Novoa et al., 2020; Pacifici et al., 2020; Parmesan & Yohe, 2003; Platts et al., 2019). Accurate descriptions of range shifts are essential to predict future trends in light of climate change (Baquero et al., 2021) and develop effective control strategies of invasive species (Uden et al., 2015). However, environmental alteration and the introduction of alien species are not the only causes of species range shifts and expansion processes. Some carnivore species are expanding their ranges following severe historical contractions, due to ceased human persecution (Chapron et al., 2014; Louvrier et al., 2018; Lubina & Levin, 1988), reduction in anthropogenic pressure on habitat (Clavero et al., 2010; Marcelli et al., 2012), or mesopredator release (LaPoint et al., 2015).

The lack of long‐term spatiotemporal data from the range of a species is a major obstacle for assessing range shifts. Monitoring programs collect high‐quality data in the form of presence‐absence (more properly, detection–non‐detection) of a species, but usually on short time scales. Thus, comparing contemporary and historical surveys is often the only way to documenting range changes, especially in native species. This may come at the cost of using past data collected using non‐standardized methods, often in the form of presence‐only data and with low or incomplete sampling effort (Tingley & Beissinger, 2009). For these reasons, quantification of species range shifts is a challenging task, requiring the use of appropriate data and range metrics for minimizing the effects of sampling errors (Hassall & Thompson, 2010; Preuss et al., 2014; Yalcin & Leroux, 2017). In some situations, sampling effort required to assess range shifts can be reduced by restricting sampling to the range margins. Then summary statistics of the distances of species occurrences from a reference location (Hassall & Thompson, 2010; Parmesan & Yohe, 2003; Preuss et al., 2014) provide a time series of relative change in the distribution limits, which can be used to quantify range expansion or contraction over time.

Detection and quantification of range shifts, and modeling range expansion are also critical steps for assessing the progress of population recovery, adjusting future conservation efforts (Marcelli & Fusillo, 2009a; Tinker et al., 2008). A limited but growing number of studies have investigated the recolonization dynamics of apex predators that have suffered human‐induced declines (Jerina & Adamič, 2008; Louvrier et al., 2018; Marcelli et al., 2012). One of the most studied cases is the range expansion of the sea otter (*Enhydra lutria*) across North Pacific following translocations (Tinker et al., 2008). Mechanistic models of ecological diffusion have been used to estimate spatiotemporal changes in occupancy and abundance of this gregarious carnivore from counts of individuals (Eisaguirre et al., 2021; Lubina & Levin, 1988; Williams et al., 2017, 2019).

Here, we used presence‐absence data from standard field surveys to assess the natural reexpansion of the Eurasian otter (*Lutra lutra*) in Italy at the northern range‐edge, over a period from 1985 to 2021. The Eurasian otter is a solitary, nocturnal mesocarnivore, living mainly in rivers and feeding on fishes and anurans (Clavero et al., 2003). The species is listed in annexes II and IV of the European Habitats Directive (92/43/EEC). After a sharp decline occurred from 1950s to 1980s, many European populations have colonized their former distribution ranges, primarily as a consequence of reduced anthropogenic pressure on rivers (Clavero et al., 2010; Marcelli et al., 2012; Marcelli & Fusillo, 2009a). In Italy, the geographic range of Eurasian otters collapsed from 1970s, mainly as a result of degradation of river habitat due to economic, industrial, and infrastructure growth of Italy in the 1960s and 1970s of the last century. Remnant populations persisted in the southern regions of the peninsula (Cassola, 1986). A comprehensive survey carried out in 2003 detected a natural recovery of the species: site occupancy increased everywhere within the borders of the former range and distribution extended toward the southernmost areas of the peninsula. A reduced impact of urban land use on river habitat was identified as a factor involved in this recovery (Marcelli & Fusillo, 2009a). Despite a significant recovery, otter populations in Italy remained confined to the southern regions of the peninsula. Accordingly, the Italian IUCN red list of vertebrate species classified the Italian population as endangered (Rondinini et al., 2013). This remnant southern population has been recognized as an evolutionary significant unit, genetically distinct from other European populations (Mucci et al., 2010). After 2003, a number of local surveys focused on range limits documented an apparent progress of the range reexpansion. While otters continued to expand southward (Marcelli & Fusillo, 2009b; unpublished data), a weak northward expansion in areas of central Italy was observed in more recent times (Lerone, 2013; Loy et al., 2015; Marcelli, M., & Fusillo, R., unpublished data). In 2019 occurrences of otters were recorded in some sites of the Pescara river, which defined the known northern limit of the distribution (Giovacchini et al., 2019).

Because of the restricted distribution, the reexpansion across the central and northern regions of the peninsula is an important piece of the conservation strategy for Italian otters (Marcelli & Fusillo, 2009a; Panzacchi et al., 2011). However, no attempt was made to compare available data and assess whether and how a northward range expansion has occurred in the last decades. Thus, the main aim of our study was to model the rate of range expansion, with a view to gaining insights into progress and potential of recovery of the otter in Italy, and mechanisms of species reexpansion. For this, we conducted an occupancy survey in 2021 and used additional data from five past surveys, to obtain a time series of the northernmost occurrences of the species. Then we used regression models in which the distance of range edge occurrences was plotted against time (year of observation) to estimate the expansion rate (Preuss et al., 2014; Wang et al., 2021).

Few studies have explicitly examined the spatiotemporal patterns of naturally reexpanding populations. Conversely, great efforts have been made to understand the processes that underlie observed patterns of biological invasions. Alien species frequently exhibit nonlinear range‐versus‐time curves. According to models of stratified diffusion, neighborhood dispersal increases density of populations during an initial phase of establishment with slow or no expansion (Johnson et al., 2006; Shigesada et al., 1995). Range expansion abruptly accelerates when density of donor populations reaches a threshold to provide sufficient numbers of long‐distance emigrants (Johnson et al., 2006; Shigesada et al., 1995). A similar mechanism could operate also in native species that reexpand their range (see Hurford et al., 2006 for reintroduced populations). Recovery of natural populations could start with an increase in density after the end of the range contraction process, with no extension in range until a threshold in density is reached at the expanding front. Thus, range reexpansion could proceed after a possibly long time lag imposed by dispersal limitation and the spatiotemporal pattern of driving factors (i.e., habitat recovery). We explored this hypothesis using site occupancy as a surrogate for density (MacKenzie & Nichols, 2004). We explicitly examined whether the range expanded abruptly after a period of range stasis using segmented regression, and compared linear and nonlinear model forms to test for an early increase in occupancy and a delayed range expansion. Our expectation is consistent with the hypothesis that low dispersal may delay colonization of distant newly suitable habitat, resulting in range limits that temporarily fall short of limits imposed by environmental gradients (Hargreaves et al., 2014; Pulliam, 2000).

## 2 METHODS

### 2.1 Study area

We delineated the study area based on data from several surveys conducted during the period 1985–2019 (Barrasso et al., 1992; Cassola, 1986; Giovacchini et al., 2019; Lerone, 2013; Loy et al., 2015; Marcelli & Fusillo, 2009a; Ottino, 1995), aiming to assess the current northern range margin of the Italian population of Eurasian otter. All these studies confirmed that the northern range margin was located in the Abruzzo region from the mid‐1980s, with the exception of one survey that reported a more southern northern limit (Marcelli & Fusillo, 2009a). We did not consider small isolated populations in more northern regions (Latium and Tuscany) gone to extinction between 1980s and 1990s (Cassola, 1986; Reggiani, G., unpublished data) and the ongoing slow recolonization of Italian Alps from other European countries (Malthieux, 2020; Pavanello et al., 2015; Righetti, 2011; Stokel et al., 2021).

**FIGURE 1:**
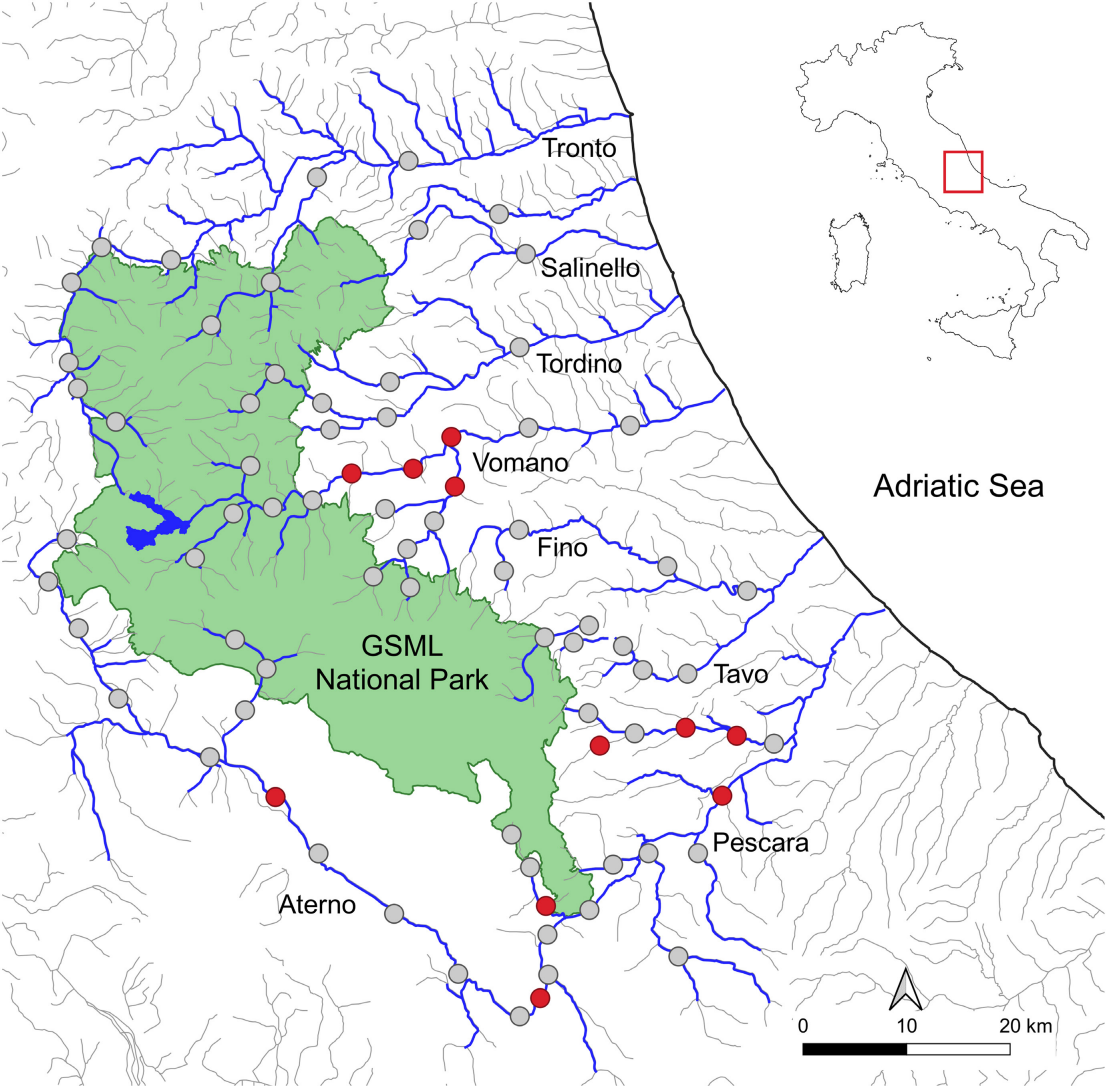
Study area located in the Abruzzo Region, Italy. Survey of the Eurasian otter performed in 2021 at the northern range periphery of Italian population: Red and gray circles are survey sites where otter was detected and not detected, respectively. Blue lines are watercourses of the survey area with Strahler stream order >1 (1:250,000‐scale hydrography layer). Green area displays the Gran Sasso and Monti della Laga National Park.

The northernmost sites of occurrences were detected in the Pescara river (central portion of Abruzzo) in 2019 (Giovacchini et al., 2019). Therefore, we included this river in our survey to delineate the southern border of the study area. The northern border was established a posteriori depending on the data that were being recorded during the survey. The resulting study area encompassed seven contiguous hydrographic basins (3696 km^2^) in the central and northern portion of the Abruzzo Region (Figure 1). About one‐third of the area is protected by the Gran Sasso and Monti della Laga National Park.

### 2.2 Field data collection

The survey was conducted during June–October 2021. We attempted to select survey sites at 5 km intervals along watercourses of the study area (Strachan & Jefferies, 1996) potentially suitable for otter occupancy. Potential habitat was defined by removing stream segments with stream order 1 (sensu Strahler, 1957) from a 1:250,000 hydrographic layer clipped to the boundaries of the survey area using QGIS 3.18.2 (QGIS Development Team, 2021). For site selection purposes, we overlaid a 5 × 5 km grid across the selected hydrographic net. Within each 5 km cell we selected a stream section based on accessibility and suitable conditions for detection of otter feces (spraints). A sampling site was a stream section of 600 m in length. To confirm otter occupancy in a site, two experienced surveyors searched for spraints, inspecting both the shorelines and all emerging structures (i.e., potential marking sites) on a full transect of 600 m if they did not detect spraints within shorter distances (Reuther et al., 2000). Data collection took place during dry seasons and days without raining events to avoid the effect of spraint washing (Fusillo et al., 2007). A total of 30 different watercourses were surveyed. All survey data were georeferenced using a GPS.

### 2.3 Range‐expansion models

We used presence‐absence data from our recent survey and five past surveys to assess a northward range expansion of Italian otters across the last four decades. Past surveys were conducted in 1985 (Cassola, 1986), 2003 (Marcelli & Fusillo, 2009a), 2012 (Lerone, 2013), 2014 (Loy et al., 2015), and 2019 (Giovacchini et al., 2019). We derive data for 1985 and 2012–2019 surveys from published maps. For analysis purposes, data from 2012 and 2014 surveys were pooled (hereafter 2012–14 data). All surveys used identical field methods but differed in geographic extension. Surveys in 1985 and 2003 were conducted across the entire peninsula and the entire otter distribution (i.e., southern regions), respectively, whereas after 2003, surveys were restricted to the northern range periphery. Spatial resolution in available data differed between surveys, consisting of EEA (European Environment Agency) 10 km cells or survey points. We georeferenced data in QGIS and resampled them to a common 10 km‐scale by overlapping the 10 × 10 km EEA grid with survey points. One point within each 10 km cell was randomly selected. Finally, we extracted centroids of the original cell data for spatial analysis.

**FIGURE 2:**
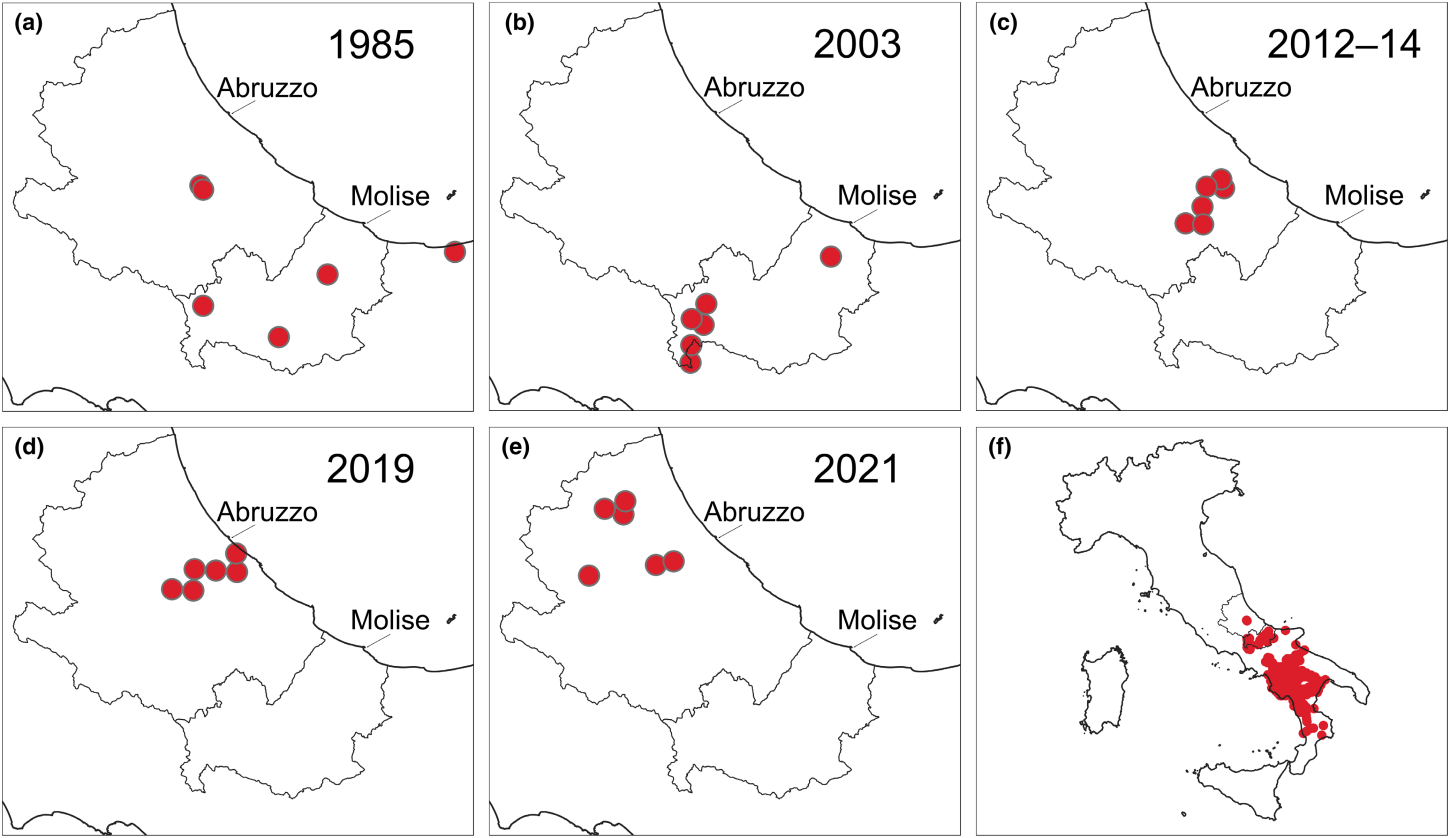
Northernmost sites of occurrence of the Eurasian otter used to quantify the northward range expansion of the Italian population across years 1985–2021 (a–e). Range‐margin occurrences were selected by five survey datasets using distances from range centers calculated for 1985 and 2003. Panel (f) shows the geographical distribution of all data of occurrence gathered from two comprehensive surveys (1985, 2003).

We selected for each survey period a sample of occurrence sites describing the northern range margin (Preuss et al., 2014). For data from surveys of 1985 and 2003, we measured the Euclidean distance of sites of occurrence from their centroid (i.e., range center). Then the northern sites above the 95th percentile of distances were selected as sites describing the northern range margin. Percentile thresholds were not applicable after 2003 because surveys were restricted to the northern range periphery. This limitation was overcome by selecting a number of northernmost sites equal to the number of sites identified in 1985, based on distances from the 1985 centroid. Then we obtained a total number of 30 range‐margin sites (six for each year) over the period 1985–2021 (Figure 2).

To quantify the northward range expansion we developed regression models relating the distance of marginal sites from the 1985 centroid (i.e., reference range center) to the year of survey (Wang et al., 2021). The regression slope provided a measure of the annual change rate (Preuss et al., 2014; Wang et al., 2021; Ward, 2005). A range reexpansion is preceded by range contraction and may be best modeled with a non‐monotonic temporal relationship. The range expansion of Italian otters at the northern margin was obvious in the recent years, but the range margin dynamics during earlier years and the onset of the expansion were not evident. We subjected data to segmented regression modeling to draw inferences about these dynamics and test for non‐monotonic patterns. Segmented regression splits the relationship between response and explanatory variable into two or more generalized linear regressions to locate points where the relationship changes (Lindeløv, 2020; Muggeo, 2008). We were interested in testing for a breaking year, where a linear increase in distance of range margin with time (i.e., range expansion) is preceded by a linear decrease or constant trend. We compared four different model forms: (1) an only‐intercept model that assumed no temporal shifts in range margin (e.g., no expansion), (2) a simple linear expansion model, (3) a quadratic model to test the hypothesis of range margin contraction followed by expansion, and two one‐breakpoint models, (4) a left‐horizontal model (i.e., the slope of the line to the left of the breakpoint is constrained to zero) to describe a stable range margin followed by expansion, (5) a two‐slope model to evaluate a variant of the hypothesis of model 3 by using two linear sections instead of an humped‐shaped curve. Models 4 and 5 estimate the year where a change in slope may take place.

Statistical analyses were performed in R 4.1.2 (R Development Core Team, 2021), using the “segmented” package (Muggeo, 2022) to run segmented models. Given a regression model, this package updates it by adding one or more segmented relationships, providing slope estimates for each segment and breakpoints where slope changes. We tested for the existence of one breakpoint using the pseudo score test (“pscore.test”) proposed by Muggeo (2016). We compared candidate models based on the second‐order Akaike information criterion (AICc) and Akaike weights (*w*_*i*_), using the AICcmodavg package (Burnham & Anderson, 2002; Mazerolle, 2020). AIC_c_ with lower values indicate better model fit and complexity (i.e., parsimony). Akaike weights represent the weight of evidence in favor of model *i* being the best model of the suite of candidate models. We calculated the difference in AIC_c_ values of alternative models relative to the model with the lowest AIC_c_ (ΔAIC_c_) to assess the relative level of support. Models with ΔAIC_c_ ≤ 2 have substantial support, whereas models where ΔAIC_c_ is greater have progressively less support.

### 2.4 Temporal models of range‐periphery occupancy

Tracking species distributional edges using only distance metrics may provide incomplete inference into the range expansion. We expected that an increase in local occupancy at the expansion front would precede the onset of range reexpansion, as an effect of low long‐distance dispersal at low density. We predicted a delay in the northward expansion of the otter range in terms of an early increase in occupancy probability coupled with no significant changes in the range edge location. We were also interested in testing an occupancy decline in recent times as otters reexpand northwards along a putative increasing gradient of anthropogenic impact. This pattern may arise when small sinks populations (Pulliam, 2000) are maintained in suboptimal habitat by dispersal from high abundance interior populations. To explore the above hypotheses, we modeled occupancy probability of otters at the northern edge as a function of year, using presence‐absence data and a Binomial distribution with a logit link. For this purpose, we extracted survey sites from minimum enclosing circles (*minimum bounding geometry* tool, QGIS 3.18.2) of the northernmost occurrences. These operations produced 126 presence‐absence sites from range of periphery areas over years 1985–2021. We evaluated the relationship of occupancy probability with time by fitting segmented, quadratic, and linear models, using the same statistical methods as those for the distance‐based analysis of range expansion.

## 3 RESULTS

### 3.1 Field survey

We surveyed 77 sites along 30 watercourses of seven river basins and found evidence of otters in 11 sites (Table 1, Figure 1). We confirmed otter's presence in the Pescara River, first detected in 2019 (Giovacchini et al., 2019) and known as the northernmost occurrence of Italian otters. We also found otter signs in two tributaries of the Pescara river (Nora and Tirino rivers) and along the Aterno river that were not previously documented as occupied by the species. Moreover, we detected new sites occupied by otters in the middle course of the Vomano river and in one tributary (Fiumetto stream) which redefine the current northern limit of distribution, about 45 km northwards of the Pescara river and in close proximity to the Gran Sasso and Monti della Laga National Park.

**TABLE 1:** River basins in the study area and numbers of watercourses, survey sites, and sites detected as occupied by otters.

River basin	Watercourses	Survey sites	Sites occupied
Aterno‐Pescara	11	30	7
Saline	3	10	0
Vomano	7	17	4
Tordino	5	7	0
Salinello	1	2	0
Vibrata	1	1	0
Tronto	2	10	0
Total	30	77	11

### 3.2 Range‐expansion models

**FIGURE 3:**
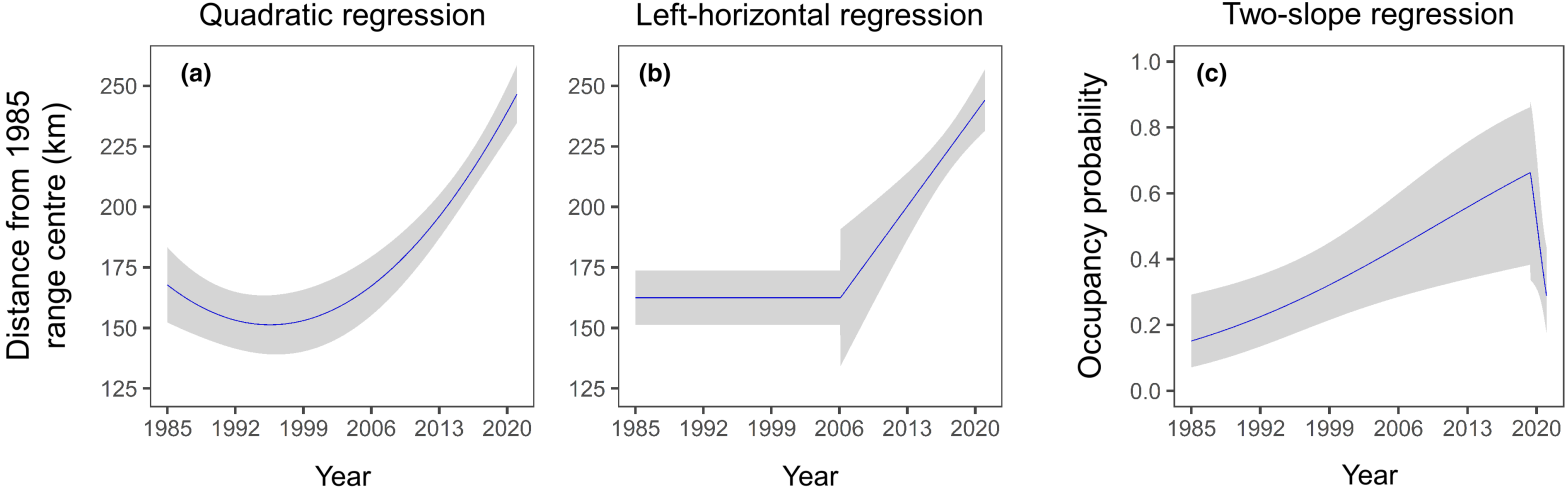
Predicted rates of range expansion and occupancy change (mean and 95% CI) at the northern range margin of the Italian population of Eurasian otter. Two regression models of distance against year were selected as equally plausible: A quadratic model (a) and a breakpoint regression model with the left slope constrained to zero (b). The best model selected for occupancy change is displayed in panel (c).

The cumulative AICc weight for the quadratic and segmented models was 1, providing evidence that the distance of northern marginal occurrences from the reference point changed across years, but not monotonically. Although the quadratic model had the highest weight of evidence (*w*_*i*_ = 0.587), it was only <2 AICc units higher than the left‐horizontal model, indicating that these two models were equally plausible (Table 2). Parameters estimates for the quadratic model (Table 3) predicted a slight decrease in distance from the reference point (i.e., range contraction) followed by an increase (i.e., range expansion) from the mid‐1990s (Figure 3). According to the left‐horizontal model (Table 3), the estimated breakpoint year occurred in 2006 (pseudo score test, *p* < .001). From that year, the distance from the reference point increased linearly with time at an average rate of 5.48 km/year (95% CI; 3.10–7.87 km/year). Thus, the northern range margin did not change between 1985 and 2006 and subsequently shifted northwards (Figure 3). The two‐slope model ranked third and produced estimates similar to those of the left‐horizontal model (Table 3). Thus, the cumulative weight of evidence for a stable range margin followed by a linear range expansion was 0.413. Despite the quadratic model predicts an initial contraction, this is only about 15 km southward. Furthermore, the upward concavity of the relationship between distance of margin locations and year relied on only two time points (1985 and 2003). Therefore, we concluded that there is no sufficient evidence that the northern margin of otter distribution changed between 1985 and 2006, and based our inference on segmented models.

**TABLE 2:** Model selection results for models of range expansion of the Eurasian otter in Italy.

Model	AIC_c_	∆AIC_c_	w_i_	−2LL	np
Quadratic	266.96	0	0.5868	128.68	3
Left‐horizontal	268.37	1.41	0.2899	129.38	3
Two‐slope	270.08	3.12	0.1233	128.79	4
Linear (no breakpoint)	288.31	21.35	0	140.69	2
Constant	309.06	42.1	0	152.31	1

*Note*: The response variable was the distance of northernmost occurrences (1985–2021) from the range center in 1985. Models included parameters for linear, quadratic, and two‐slope linear (segmented) regressions of distance on year (Gaussian family). In the left‐horizontal model the left slope was constrained to zero. Given are the relative difference in AICc (second‐order Akaike information criterion) compared to the top ranked model (∆AICc), the AICc weights (*w*_*i*_), the negative of twice the logarithm of the likelihood function evaluated at the maximum likelihood estimates (−2LL) and the number of parameters (np).

**TABLE 3:** Parameter estimates for models of range expansion of the Eurasian otter in Italy.

Model	Parameter	Mean	SE	95% CI lower	95% CI upper
Quadratic	Intercept	167.78	7.56	152.27	183.29
	Year	−588.14	102.544	−798.54	−377.74
	Year^2^	0.1474	0.0256	0.0948	0.1999
Left‐horizontal	Intercept	162.46	5.496	151.69	173.23
	Year 1	–	–	–	–
	Breakpoint year	2006.1	2.709	2000.5	2011.6
	Year 2	5.48	1.163	3.10	7.87
Two‐slope	Intercept	168.09	7.77	152.13	184.05
	Year 1	−0.62	0.901	−1.88	0.63
	Breakpoint year	2004.9	2.854	1999.0	2010.7
	Year 2	6.11	1.279	3.10	7.87
Linear	Intercept	149.46	10.05	128.88	170.04
	Year	2.17	0.379	1.39	2.95
Constant	Intercept	199.4	7.202	184.66	214.12

*Note*: The response variable was the distance of northernmost occurrences (1985–2021) from the range center (1985). Models included parameters for linear, quadratic and two‐slope linear (segmented) regressions of distance on year (Gaussian family). In the left‐horizontal model the left slope was constrained to zero. Given is the estimated breakpoint year where the linear relationship changes.

### 3.3 Temporal models of range‐periphery occupancy

The two‐slope segmented regression was the most parsimonious model for the trend in occupancy probability at the northern range edge across years (Table 4). AIC metrics showed that the relative likelihood of this model was 4.4 times higher (*w*_*i*_ = 0.733) than the quadratic model (*w*_*i*_ = 0.169), which ranked second (ΔAIC_c_ = 2.9). The remaining models received virtually no support. According to the model selected, a significant breakpoint (pseudo score test, *p* = .044) in occupancy probability occurred around 2019, indicating an abrupt transition from an increasing trend to a steeper decreasing one (Figure 3). The comparison between years indicated that occupancy probability was lowest in 1985 (0.151, CI = 0.072–0.292), then increased substantially in subsequent years up to 0.658 (95% CI = 0.857–0.381) and strongly declined in 2021 (0.289, CI 0.436–0.176).

**TABLE 4:** Model selection results for models of changes in range‐periphery occupancy of the Eurasian otter in Italy.

Model	AICc	∆AICc	w_*i*_	−2LL	np
Two‐slope	151.78	0	0.7330	71.73	4
Quadratic	154.72	2.94	0.1685	74.26	3
Linear (no breakpoint)	156.59	4.81	0.0662	76.25	2
Left‐horizontal	159.30	7.52	0.0171	76.55	3
Constant	159.52	7.74	0.0153	78.74	1

*Note*: The response variable was presence‐absence of otters at the northern range periphery (1985–2021). Models included parameters for linear, quadratic, and two‐slope linear (segmented) regressions of site occupancy probability on year (binomial family, logit link function). In the left‐horizontal model, the left slope was constrained to zero. Given are the relative difference in AICc (second‐order Akaike information criterion) compared to the top ranked model (∆AICc), the AICc weights (*w*_*i*_), the negative of twice the logarithm of the likelihood function evaluated at the maximum likelihood estimates (−2LL) and the number of parameters (np).

## 4 DISCUSSION

We modeled changes in northern range edge of Italian otters over a 36‐year time period using presence–non‐detection data. We detected new northernmost occurrences in 2021 by field survey, and analyzed additional data collected at the northern range periphery from 1985 to 2019. Using segmented regressions of distance and occupancy measures on year, we were able to identify a northward range expansion with three distinct phases. From 1985 to 2006, no expansion was detected, but site occupancy at the northern range border was predicted to increase. From 2006 to 2019, the northern range edge shifted northwards and edge occupancy probability continued to increase. Between 2019 and 2021, the northern edge continued to shift northwards, while occupancy abruptly declined. Here, we review the assumptions and limitations of the study and discuss the potential mechanisms underlying the inferred patterns. We conclude with implications for otter conservation and future monitoring.

### 4.1 Study limitations

Although using historical data is often the only way to infer changes in species range, a problem arises from the comparability of surveys with different underlying properties (Tingley & Beissinger, 2009). This could be a potential source of bias in our study. However, the nature of data and the methods employed should have minimized this problem. All surveys from which we obtained data used the same methodology. In addition, we reduced differences in sampling effort among the original data by aggregation on the 10 km grid. On the other hand, sampled areas were not identical. After the nationwide survey of 1985, subsequent surveyors set the northern margin of the sampled area about 10–40 km from the new northernmost occurrences recorded. This procedure produced sampled areas that slightly shifted northward over years, following the observed range expansion of otters, but at the same time reduced the possibility of not sampling the current limit of distribution. Therefore, it seems reasonable to assume that the difference in sampled area was not a significant source of bias.

Even when survey characteristics and methods are similar, imperfect detection probability may differ between sampling periods leading to biased inferences about range dynamics (Guillera‐Arroita, 2017; Tingley & Beissinger, 2009). The severity of the problem depends on the level of detectability of a given species and largely on the observer skill. A powerful tool to account for imperfect detection in the modeling of species distribution is the occupancy modeling framework (MacKenzie et al., 2003). Fundamental to this framework is the collection of multiple detection–non‐detection records obtained by repeat visits or spatial sub‐sampling of survey sites (MacKenzie & Royle, 2005). The framework can extend to studying species' range dynamics (Rushing et al., 2019; Tingley & Beissinger, 2009). Unfortunately, available data for modeling range dynamics often are not part of designed studies, but largely come from historical data that lack information about imperfect detection. This was also the case for the data used in our analysis. We are aware that our study may suffer from unmodeled detection probability. However, all data were collected through an efficient sampling protocol, involving standardized searching of otter's feces along 600 m transects, which has shown to detect the presence of otters with relative high probability (Fusillo et al., 2007; Marcelli et al., 2012), especially when experienced surveyors are involved (Jeffress et al., 2011). This requirement is largely satisfied in our dataset. Data from 2003 onwards originate from few otter experts. Observers involved in the 1985 survey had less expertise, but were specifically trained to otter surveys (Cassola, 1986). Therefore, we are confident that field methods and the intrinsic detectability of otter markings minimized potential biases from imperfect detection.

Distance measures that do not model explicitly detection probability have been traditionally used to assess species range shifts (Hickling et al., 2005; Parmesan & Yohe, 2003; Pöyry et al., 2009; Thomas & Lennon, 1999). Simulation studies have shown that the gamma quantile method for range margin description is the best choice in many sampling scenarios (Hassall & Thompson, 2010, but see Bates et al., 2015). Simple descriptive statistics based on distances of the most extreme occurrences perform well when sampling effort focuses on range margins (Hassall & Thompson, 2010; Preuss et al., 2014). Consequently, we applied this type of descriptive metric to the data. Despite some limitations, our dataset provided a rare opportunity to explore hypotheses about patterns in range expansion of a recovering species over a long time period.

### 4.2 Patterns of range expansion

We were specifically interested in quantifying the northward range expansion of otters and identifying the timing of onset of this process. This required data covering a large enough temporal window to capture information prior to expansion and fitting non‐monotonic models. By using segmented regression we considered a model with a sharp transition between no directional change in range margin and subsequent expansion at a constant rate. The segmented model indicated that otters extended their range northward from 2006, with a significant increase in distance of the northernmost occurrences from the reference range center and a mean slope of 5.48 km/year. From 2006 to 2021, the overall northward shift was about 80 km.

The prolonged stability of the northern range margin suggests two possible scenarios. The first is that the distribution of the otter toward and beyond the northern border of the range reflected its environmental requirements. In other words, the range limit was imposed by the equilibrium of the species with the distribution of environmental suitable conditions (Sexton et al., 2009). However, for many species equilibrium conditions are unlikely, because gradients in habitat quality and quantity are always changing over time (Yackulic et al., 2015). This may be especially true when the range edge stability is protracted over long time, as in our study. Therefore, we considered a second scenario where low dispersal restricts colonization beyond range edge. We analyzed changes in site occupancy to explore this hypothesis. Our best model quantified a significant increase in occupancy during the phase of stable range margin. Indeed, occupancy of edge populations was only 0.15 in 1985 and was predicted to increase to about 0.4 in 2006, when otters started to expand their range.

Theoretical models of species invasion explained range expansions preceded by periods of range stasis as an interaction between Allee effects (Stephens et al., 1999) and low long‐distance dispersal (Johnson et al., 2006). Long‐distance dispersal can produce range expansions only when edge populations have grown large enough to provide sufficient numbers of emigrants beyond the range border (Shigesada et al., 1995). If dispersal is too low to inflate the density of nascent populations above an Allee threshold (the minimum density required for a population to grow), then range expansion fails (Johnson et al., 2006). A similar mechanism could also operate in species that expand their ranges after human‐induced contractions. The long stasis in range margin and the concurrent occupancy increase that we observed in otters seem to support this hypothesis. The position and the very low occupancy of the northern edge in 1985 probably approximated the endpoint of the range contraction process toward the southern areas of Italy. During the early phase of the recovery, neighborhood colonization could have increased northern edge occupancy up to about 0.4. Then the 0.4 value may have likely acted as a threshold above which increased long‐distance dispersal allowed range expansion to start and progress in the later phase from 2006 onwards. We think that the environmental changes that drove range expansion in otters were also involved in the initial increase in occupancy. If this was the case, the northern range limit in the stasis phase was not in equilibrium with environmental conditions and mainly constrained by dispersal lag. An alternative to dispersal lag explanation for stable range edge is that otter population tracked changing conditions perfectly through time and space, with driving factors acting locally in the long initial phase and spreading rapidly northward in subsequent times. However, such view is over‐simplified and less realistic, although it is possible that favorable conditions spread northward and played a role. Several human‐related changes could have plausibly driven the reoccupation of northern habitat again suitable for otters. Most of these factors were probably related to a reduction of human pressure on river habitat. Human development pressure during the rapid economic and industrial growth of Italy in the 1960s and 1970s of the last century was the ultimate cause of range contraction of the otter in Italy (Marcelli & Fusillo, 2009a).

Occupancy at the range edge further increased up to 0.66 in 2019, but dropped to 0.29 in 2021. The nature of the most northward occurrences detected in 2021 in the northern Abruzzo region is uncertain. The low occupancy may suggest a range‐edge population acting as demographic sink (Pulliam, 2000), maintained in unsuitable habitats by dispersal from interior populations. On the other hand, the time interval between the last two surveys (2 years) indicates a recent colonization, suggesting the possibility of a self‐sustaining edge population able to increase in abundance and occupancy.

### 4.3 Conservation implications

Italian otters extended their range southward about 110 km from 1985 to 2003 (Marcelli & Fusillo, 2009a) and continued to expand in this direction in the following years (Marcelli & Fusillo, 2009b; unpublished data). Thus, range extension was smaller (about 80 km) and much more recent into northern areas than into southern areas. This may suggest a stronger resistance of northern Italian landscapes to colonization by otters due to less suitable habitat and potential barriers. Heterogeneity in landscape structure and resource availability strongly affect expansion rates across space (Fraser et al., 2015; Hastings et al., 2005; Veech et al., 2011). Asymmetrical spatial patterns have been also observed in other native mesocarnivores (Sainsbury et al., 2019).

Although recovering otters have shown a high flexibility in their habitat selection (Weinberger et al., 2016), the strong decline in occupancy observed at the current northern range edge of Italian otters may reflect a deterioration in habitat conditions that could slow down further range reexpansion toward the northern areas. Indeed some evidence suggests a decreasing habitat suitability from the interior areas toward the recent northern periphery of the range. The mean stream gradient (i.e., the slope of the stream's channel) of main watercourses is about two‐fold higher in the current than in the former northern range periphery (south Abruzzo and Molise regions) due to mountain topography. This means that otters in the new colonized areas cope with watercourses on average steeper and more oligotrophic than in the interior range areas, with high‐energy demand for moving and lower resource availability. With the exception of the Gran Sasso and Monti della Laga National Park, many rivers, especially their lower reaches with potentially high fish availability, are impacted by industrial and commercial areas, roads, water pollution, and sand‐gravel extraction (Vomano and Aterno rivers, Maran, 2004; Paolini et al., 2005). In general, human population density is higher in the current than in the former northern range periphery (122 inhab/km^2^ vs. 69 inhab/km^2^). Moreover, main watercourses are disturbed by dams and water diversion for energy production. In particular, the Vomano river and its tributaries are severely impacted by five large dams (Maran, 2004). Three of them are between 40 and 50 m high, built in narrow gorges with steep rocky walls, and therefore could be difficult to pass for otters. Therefore, although encouraging range expansion is in theory a relevant piece of the strategy for long‐term conservation of Italian otters, it is a challenging task that requires active conservation and river ecosystem restoration. Highly focused management in National Parks and in the network of riverine Special Areas of Conservation designated under the European Habitats Directive (92/43/EEC), could be a great opportunity to achieve successful conservation outcomes. Essential is the attainment of agreements between protected areas, regional governments, and hydropower companies, aimed at reducing or mitigating the impacts of water diversion, hydropeaking, and dams on river ecosystems. The designation of new Natura 2000 sites (92/43/EEC) along watercourses with high potential for otter colonization may enhance this strategy. Moreover, the environmental agenda of Abruzzo and Marche regions under the current (2021–2027) European Regional Policy funding, can be another opportunity for river and otter conservation in these critical areas. The Gran Sasso National Park can play a leading role in this process of active conservation and habitat restoration, also in the view of a likely otter colonization in the immediate future. The designation of buffer zones to maximize the cumulative extension of middle reaches of rivers under protection may greatly encourage further habitat recolonization of Italian otters.

## 5 CONCLUSIONS

We first documented the presence of the otter into the Continental biogeographical region of Italy, suggesting a new monitoring task for conservation assessment under Articles 11 and 17 of the European Habitats Directive. A monitoring program can also inform habitat management aiming at encouraging the northward colonization. We strongly advocate the systematic collection of detection–non‐detection data and a study‐design framework for dynamic occupancy analysis (Guillera‐Arroita, 2017). This involves recording repeated observations at the same sampling sites for each survey season to deal with imperfect detection of otters (Fusillo et al., 2007; Marcelli et al., 2012), allowing more accurate inference of range expansion. Our results can be used as pilot data for designing future monitoring of the northern range periphery of Italian otters. Based on our estimate of range expansion rate and a time period of 10 years, we recommend a study area extended at least 60 km in both north and south directions from the current northernmost occurrence of otters.

## AUTHOR CONTRIBUTIONS

**Manlio Marcelli:** Conceptualization (equal); formal analysis (equal); investigation (equal); methodology (equal); writing – original draft (lead); writing – review and editing (equal). **Federico Striglioni:** Conceptualization (supporting); writing – review and editing (equal). **Romina Fusillo:** Conceptualization (equal); formal analysis (equal); investigation (equal); methodology (equal); writing – review and editing (equal).

## CONFLICT OF INTEREST

Authors declare no conflicts of interest.

## Data Availability

Data are available on Dryad at https://doi.org/10.5061/dryad.2280gb5ww.
